# Data-Driven Calibration of Soil Moisture Sensor Considering Impacts of Temperature: A Case Study on FDR Sensors

**DOI:** 10.3390/s19204381

**Published:** 2019-10-10

**Authors:** Liping Chen, Lili Zhangzhong, Wengang Zheng, JingXin Yu, Zehan Wang, Long Wang, Chao Huang

**Affiliations:** 1 National Research Center of Intelligent Equipment for Agriculture, Beijing 100097, China; chenlp@nercita.org.cn (L.C.); lilizhangzhong@163.com (L.Z.); zhengwg@nercita.org.cn (W.Z.); yujx@nercita.org.cn (J.Y.); 2 Key Laboratory for Quality Testing of Hardware and Software Products on Agricultural Information, Ministry of Agriculture, Beijing 100097, China; 3 School of Computer and Communication Engineering, University of Science and Technology Beijing, Beijing 100083, China; 15233751966@163.com; 4 Key Laboratory of Wind Energy and Solar Energy Technology (Inner Mongolia University of Technology), Ministry of Education, Hohhot 010051, China

**Keywords:** calibration, data-driven, impacts of temperature, soil moisture sensor

## Abstract

Commercial soil moisture sensors have been widely applied into the measurement of soil moisture content. However, the accuracy of such sensors varies due to the employed techniques and working conditions. In this study, the temperature impact on the soil moisture sensor reading was firstly analyzed. Next, a pioneer study on the data-driven calibration of soil moisture sensor was investigated considering the impacts of temperature. Different data-driven models including the multivariate adaptive regression splines and the Gaussian process regression were applied into the development of the calibration method. To verify the efficacy of the proposed methods, tests on four commercial soil moisture sensors were conducted; these sensors belong to the frequency domain reflection (FDR) type. The numerical results demonstrate that the proposed methods can greatly improve the measurement accuracy for the investigated sensors.

## 1. Introduction

The determination of soil moisture content is of great importance to the management of agriculture and in the field of hydrological engineering [1,2]. In modern agriculture, soil moisture is frequently monitored to better schedule irrigation [3,4]. A variety of approaches have been developed to measure soil moisture content based on various techniques including the thermo-gravimetric technique, the calcium carbide technique, the neutron scattering technique, dielectric techniques, electrical impedance sensors, and the thermal dissipation block technique [5,6,7]. These techniques differ in measuring principles, accuracy, and complexity. The thermo-gravimetric technique measures the soil moisture content by drying the soil sample in an oven. This technique can provide accurate measurements of soil moisture and is often used as the standard reference. However, thermo-gravimetric-technique-based measurement is time-consuming and requires special equipment, which constrains the application of such a technique, especially for in-situ measurements. Other techniques employ the physical and chemical properties of soil to measure its moisture content.

Dozens of commercial soil moisture sensors have been developed based on these techniques; however, the difference between the measured and actual soil moisture is often observed, and the accuracy of these sensors varies due to the employed techniques as well as the working conditions such as the operating temperature. To improve the measurement accuracy, calibration of the soil moisture sensor is required, including sensor-specific calibration and site-specific calibration [8,9]. The former conducts direct calibration between sensor response and soil moisture content, while the latter takes the soil texture variation into account. In [10], a sensor-specific procedure based on reference media is proposed for the calibration of low-cost soil water content sensors. In literature, polynomial equations are widely applied to calibrate soil moisture sensors [11,12]. Since polynomial equations are of fixed forms, their capability of reflecting the complex relationship between the actual and measured soil moisture is limited. Meanwhile, some environmental factors, especially the temperature, are normally ignored during the calibration [10]. Recently, efforts have been made to understand and compensate the temperature impacts on measurement accuracy [13,14].

In this paper, a pioneer study on the data-driven calibration of soil moisture sensors was investigated. To our best knowledge, it is the first time that data-driven methods have been applied into the calibration of soil moisture sensor. Two data-driven models including the multivariate adaptive regression splines (MARS) [15,16] and the Gaussian process regression (GPR) [17,18] were developed to calibrate soil moisture sensor considering the impacts of temperature on the accuracy of measurements. Compared with the conventional polynomial models, MARS is a nonparametric regression technique and can effectively model the nonlinearities and interactions between variables, while the GPR is a kernel-based regression model. The efficacy of the proposed methods were verified on different commercial soil moisture sensors.

The remainder of the paper is organized as follows: Section 2 describes the analysis of the impact of temperature on measurement accuracy based on four commercial soil moisture sensors. The calibration methods developed based on data-driven models considering temperature impacts are described in Section 3, which is followed by numerical experiments in Section 4. Conclusions are drawn in Section 5.

## 2. Analysis of Temperature Impacts on Measurement Accuracy

This study was based on four commercial soil moisture sensors indexed as A, B, C, and D. These sensors are widely used in China. The four sensors belong to the frequency domain reflection (FDR) type, which measures the soil moisture through electronic constants. The specifications of the sensors are summarized in Table 1.

In this study, the soil from the tillage layer (0–20 cm) was used for the experiments, and its parameters of characteristics are given in Table 2. The procedures of the experiments composed of the following steps: (1) The soil sample was dried and sieved with a 2 mm sieve; (2) the wet soil samples with different levels of soil moisture content such as 9.58%, 18.25%, and 27.01% were obtained by applying the soil mixing method; and (3) the readings of the sensors were recorded at considered temperatures (0, 5, 10, 15, 20, 22, 25, 30, 35, 40, 45 °C) in temperature-controlled chambers. In the experiment, the samples were sealed with film to avoid the impact of water evaporation. The considered test temperatures fall in the range of the environmental temperature during the growth of wheats and corns in Northern China. The standard Gravimetric technique was applied to obtain the actual soil moisture [19]. The soil samples were dried in the oven at 105°C for 48 h to a constant weight before the soil moisture was computed.

The size of test data points for each sensor was 70. Examples of records of operating temperature (*T*), sensor reading, and actual (reference) soil moisture content are illustrated in Table 3. From Table 3, there are errors on the measurements of soil moisture content by the sensor, and the sensor reading can be quite different under various operating temperatures.

**Figure 1:**
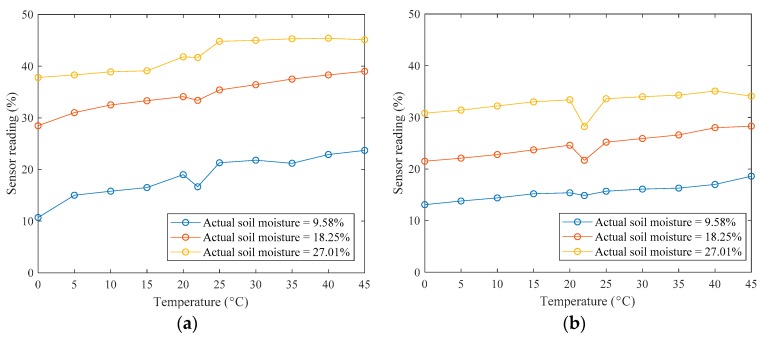

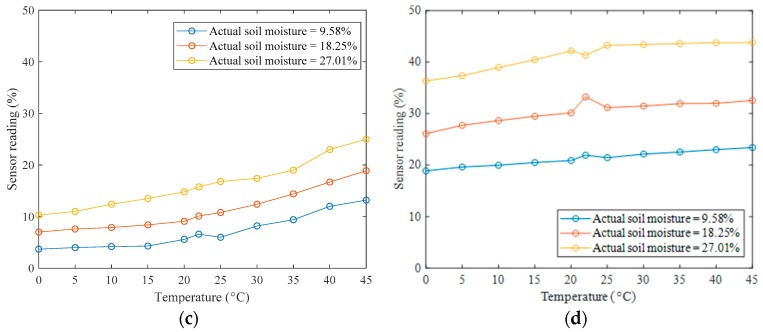
Illustration of sensor readings at various temperatures: (**a**) sensor A; (**b**) sensor B; (**c**) sensor C; and (**d**) sensor D.

To better illustrate the temperature impacts on the measurements, the sensor reading at various temperatures for different levels of actual soil moisture content is illustrated in Figure 1. It is observable that the sensor reading tends to increase with increasing temperature, while the trend of variation highly depends on the level of actual soil moisture content and the type of soil moisture sensor. This is partially due to the increasing temperature strengthening the polarization of the soil and the movement of water molecules, resulting in a larger soil dielectric constant.

From above analysis, it is valuable to investigate the calibration methods of soil moisture sensors considering the impact of temperature.

## 3. Methodology

**Figure 2:**
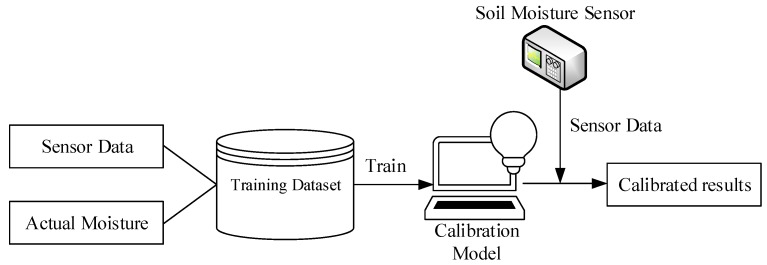
The framework of the proposed data-driven calibration method.

The framework of the proposed data-driven calibration methods is depicted in Figure 2, which consists of the following four steps:

(1) Prepare the training dataset: Collect both the soil moisture sensor data and the actual soil moisture content via experiments.
(2) Develop the calibration model: Train a regression model based on the multivariate adaptive regression splines (MARS) and Gaussian process regression (GPR) algorithms on the training dataset.
(3) Model evaluation: Compute the calibration errors using the learned model and the test dataset.
(4) Model application: Collect new sensor data and apply the calibration model to yield the calibrated soil moisture content.

The development of data-driven calibration methods based on the MARS and the GPR models was introduced as follows.

### 3.1. Multivariate Adaptive Regression Splines

The MARS model was built to generate more accurate soil moisture content from the original sensor readings. Given the original sensor reading *r*, the calibrated soil moisture *y* was yielded according to Function (1):

(1) y=f(r,T)

where *T* is the environmental temperature and *f* is the MARS model. The MARS model is described as [15]:

(2) $$f(x)=\sum_{i=1}^{k}c_{i}B_{i}(x)$$

where **x** = [*r,T*], *c_i_* is a constant value, and *B_i_*(**x**) is a basis function. In the MARS model, the basis function includes the constant, hinge function, and product of two or more hinge functions. Two types of hinge functions, **max**(0, **x**-**constant**) and **max**(0, **constant**), were considered in the MARS model.

The MARS model was trained by minimizing the loss function in Function (3) on the training data set. The forward and the backward pass procedures [15] as well as the generalized cross-validation were applied to avoid over-fitting, and thus a more robust model was obtained.

(3) $$\min\sum_{i=1}^{n}{\left(y_{i}-f\left(x_{i}\right)\right)}^{2}$$

### 3.2. Gaussian Process Regression

To improve the accuracy of soil moisture content measurement, a GPR model was applied to capture the relationship among the operating temperature *T*, original soil moisture sensor reading *r*, and actual (reference) soil moisture content as in (4) [17]:

(4) y=f(x)+ζ

where *y* represents the actual (reference) soil moisture content, while **x** = [*r*, *T*], and *ζ* ~ *N*(0, *δ*^2^). In Equation (4), *f*(**x**) are latent functions from a Gaussian process, which is a collection of random variables, and any finite number of such variables follows a joint Gaussian distribution. A Gaussian process is specified by its mean function *m*(**x**) and its covariance function *g*(**x**, **x**′) as $f\left(x\right)\sim GP(m(x),g(x,x^{\prime }))$. In practice, the data are generally normalized to have a zero mean.

Consider the training data set *D* = (**X**, **y**), where $X=\left\{x_{i},x\in R^{d}{|}_{i=1}^{n}\right\}$ denotes the samples of predictor and $y=\left\{y_{i},y\in R|{}_{i=1}^{n}\right\}$ denotes the samples of response. In Equation (4), the response *y* results from additive combination of Gaussian variables *f*(**x**) and *ζ*; hence, *y* also follows a Gaussian distribution. The GPR model associated to the training data set can be expressed as:

(5) $$y\sim N\left(0,G\left(X,X\right)+\delta^{2}I\right)$$

where *G*(**X**, **X**) denotes the covariance matrix with *G_ij_* = *g*(**x***_i_*, **x***_j_*) and **I** is an identity matrix. The Gaussian kernel (6) is frequently considered in the applications of GPR models:

(6) $$g\left(x_{i},x_{j}\right)=\sigma_{f}^{2}\exp\left(-\frac{1}{2}\left(x_{i}-x_{j}\right)M{\left(x_{i}-x_{j}\right)}^{T}\right)$$

In Equation (6), the $\sigma_{f}^{2}$ is the signal variance, and the diagonal matrix, **M** = *diag* [1/$\lambda_{1}^{2}$, 1/$\lambda_{2}^{2}$, …, 1/$\lambda_{d}^{2}$], contains the length scales of the process. Parameters **θ** = {*δ*^2^, **M**, $\sigma_{f}^{2}$} of the GPR model are derived by maximizing the logarithm marginal likelihood function based on the training data set as in Equation (7).

(7) $$\max\log p(y|X,\theta )=\max(-\frac{1}{2}\log\left|G\right|-\frac{1}{2}{\left(y-0\right)}^{T}G^{-1}(y-0)-\frac{n}{2}\log(2\pi ))$$

For a test point $x_{*}$, the joint distribution of the response $y_{*}$ associated to the training data set follows:

(8) $$\left[\begin{array}{c} y\\ y_{*} \end{array}\right]\sim N\left(0,\left[\begin{array}{cc} G\left(X,X\right)+\sigma^{2}I & G\left(X,x_{*}\right)\\ G\left(x_{*},X\right) & g\left(x_{*},x_{*}\right) \end{array}\right]\right)$$

According to the theory of the joint Gaussian distribution, the predictive distribution of $y_{*}$ is written as:

(9) $$y_{*}|X,y,x_{*}\sim N\left(\mu ,\sum \right)$$

where $\mu =G\left(x_{*},X\right){\left[G\left(X,X\right)+\sigma^{2}I\right]}^{-1}y$ and $\sum =g\left(x_{*},x_{*}\right)-G\left(x_{*},X\right){\left[G\left(X,X\right)+\sigma^{2}I\right]}^{-1}G\left(X,x_{*}\right)$.

The prediction of the response was assumed to be:

(10) $${\hat{y}}_{*}=\mu \;$$

## 4. Computational Experiments and Results

### 4.1. Performance Metrics

In this study, error metrics including mean bias error (MBE), mean absolute error (MAE), and root mean square error (RMSE) were employed to verify the effectiveness and efficiency of the proposed methods in improving the accuracy of soil moisture content measurements by considering the impacts of temperature:

(11) $$MBE=\frac{1}{n}\sum_{i=1}^{n}{\hat{y}}_{i}-y_{i}$$

(12) $$MAE=\frac{1}{n}\sum_{i=1}^{n}\left|{\hat{y}}_{i}-y_{i}\right|$$

(13) $$RMSE=\sqrt{\frac{1}{n}\sum_{i=1}^{n}{\left({\hat{y}}_{i}-y_{i}\right)}^{2}}$$

where $\hat{y}$ and *y* indicate the modeling and actual soil moisture content, respectively, and *n* is the number of test data points.

### 4.2. Experiment Results

The proposed calibration models were developed for each device. The size of available data points was small. To comprehensively verify the efficacy of the proposed methods on the entire data set, the cross-validation technique was applied to implement the proposed methods using the following steps:

(Step 1) The entire data set *D* is randomly partitioned into *k* folds, *D* = {*D*_1_, …, *D_k_*};

(Step 2) Train a MARS/GPR model with data set *P_i_* = *D*\*D_i_*, which is the complementary data set of *D_i_*, for *I* = 1, …, *k*;

(Step 3) Apply the MARS/GPR model in Step 2 to predict the soil moisture content on the data set *D_i_* for *I* = 1, …, *k*;

(Step 4) Calculate modeling errors on the entire data set *D*.

Table 4 illustrates one set of GPR model parameters for test sensors, while the prediction equations of MARS model are given in Equations (14)–(17).

Sensor A:

(14) $$\begin{array}{c} y=0.0954-1.8543F(r|-1,\;0.205,\;0.22,\;0.2665)-0.0091F(T|+1,\;18.5,\;22,\;23.5)\\ \;+1.2013F\left(r|+1,\;0.2665,\;0.313,\;0.352\right)\;+\;0.0072F(T|-1,23.5,25,35)+1.6958F(r|\\ \;-1,0.1485,0.19,0.205)\\ -0.0832F(r|-1,\;0.352,\;0.391,\;0.4394)+0.0072F(T|+1,\;7.5,\;15,\;18.5)-0.0057F(T|\\ \;-1,\;7.5,\;15,\;18.5) \end{array}$$

Sensor B:

(15) $$\begin{array}{c} y=0.1339-0.7589F(r|+1,\;0.1585,0.163,0.2445)-0.0386F(T|+1,21,22,\;23.5)\\ \;-0.02774F(T|+1,17.5,20,21)\\ \;+0.0128F(T|+1,\;23.5,\;25,\;35)-0.7369F\left(-1,\;0.331,\;0.336,\;0.3912\right)+0.5392F(r|\\ \;-1,\;0.2445,\;0.326,\;0.331)\\ \;+1.5544F(r|+1,\;0.1425,\;0.154,\;0.1585)-0.0034F(T|+1,\;7.5,\;15,\;17.5)\\ \;+0.0008F(T|-1,7.5,\;15,\;17.5) \end{array}$$

Sensor C:

(16) $$\begin{array}{c} y=0.0883-0.0044F(T|-1,\;21,\;22,\;33.5)-0.0029F(T|\\ \;-1,\;7.5,\;15,\;17.5)+2.7210F(r|+1,\;0.047,\;0.057,\;0.0625)\\ -7.0325F(r|-1,\;0.047,\;0.057,\;0.0625)+3.1351F(r|-1,\;0.0625,\;0.068,\;0.081)\\ -1.1224F(r|+1,\;0.081,\;0.094,\;0.172)-0.0064F(T|+1,\;17.5,\;20,\;21)\\ \;+0.0098F(T|-1,\;17.5,\;20,\;21) \end{array}$$

Sensor D:

(17) $$\begin{array}{c} y=0.1066-0.2902F(r|-1,\;0.29955,0.3737,0.40195)+0.0020F(T|\\ \;+1,\;21,22,\;23.5)\\ \;+0.2107F(r|-1,\;0.40195,\;0.4302,\;0.5105)-0.0027F(T|\\ \;+1,\;10,\;20,\;21)+0.0018F(T|-1,\;10,\;20,\;21)\\ \;+0.8319F(r|+1,\;0.2071,\;0.2254,\;0.29955)-1.2090F(r|-\\ \;1,\;0.2071,\;0.2254,\;0.29955) \end{array}$$

In Equations (14)–(17), the basis function is defined in Functions (18) and (19), where *x* is the input variable being either *r* (moisture sensor reading) or *T* (temperature):

(18) $$F(x|s=+\;1,c_{-1},c,c_{+})=\{\begin{array}{cc} 0 & \;\mathrm{for}\;x\leq c_{-}\\ p_{+}{\left(x-c_{-}\right)}^{2}+q_{+}{\left(x-c_{-}\right)}^{3} & \;\mathrm{for}\;c_{-}<x\\ x-c & \;\mathrm{for}\;x\geq c_{+} \end{array}<c_{+}$$

(19) $$F(x|s=-1,c_{-1},c,c_{+})=\{\begin{array}{cc} -\left(x-c\right) & \;\mathrm{for}\;x\leq c_{-}\\ p_{-}{\left(x-c_{+}\right)}^{2}+q_{-}{\left(x-c_{+}\right)}^{3} & \;\mathrm{for}\;c_{-}<x\\ 0 & \;\mathrm{for}\;x\geq c_{+} \end{array}<c_{+}$$

with:

$p_{+}=(2c_{+}+c_{-}-3c)/{\left(c_{+}-c_{-}\right)}^{2}$
$q_{+}=(2c-c_{+}-c_{-})/{\left(c_{+}-c_{-}\right)}^{3}$
$p_{-}=(3c-2c_{-}-c_{+})/{\left(c_{-}-c_{+}\right)}^{2}.$

To illustrate the effectiveness of the proposed calibration methods considering the impacts of temperature, the performance of the proposed methods was compared with the sensor reading as well as data-driven methods developed only using information from the original sensor reading.

The comparison of modeling performances by different methods are provided in Table 5. It is observed from Table 5 that large differences exist between the measured and actual soil moisture data. More accurate soil moisture was obtained by using the data-driven calibration methods in terms of MBE, MAE, and RMSE. Moreover, the consideration of temperature impacts highly improved the modeling accuracy with the data-driven models. Therefore, the MARS model and the GPR model are effective for developing the data-driven calibration method for soil moisture sensors considering temperature impacts. Between the MARS model and the GPR model, neither dominated the other for all three metrics and four sensors.

Table 6 illustrates an example of the modeling performance at various temperatures. It is observable that at the extremely high and low temperature, the improvement of modeling accuracy by incorporating the temperature information was much greater than by only using the sensor reading. In China, the ambient temperature can be around 0 °C during the growth period of winter wheat, while the ambient temperature can be greater than 35 °C during the growth period of summer corn. Hence, the improvement of measurement accuracy at high/low temperatures can be of great importance to the arrangement of irrigation during the growth period of crops in different seasons.

**Figure 3:**
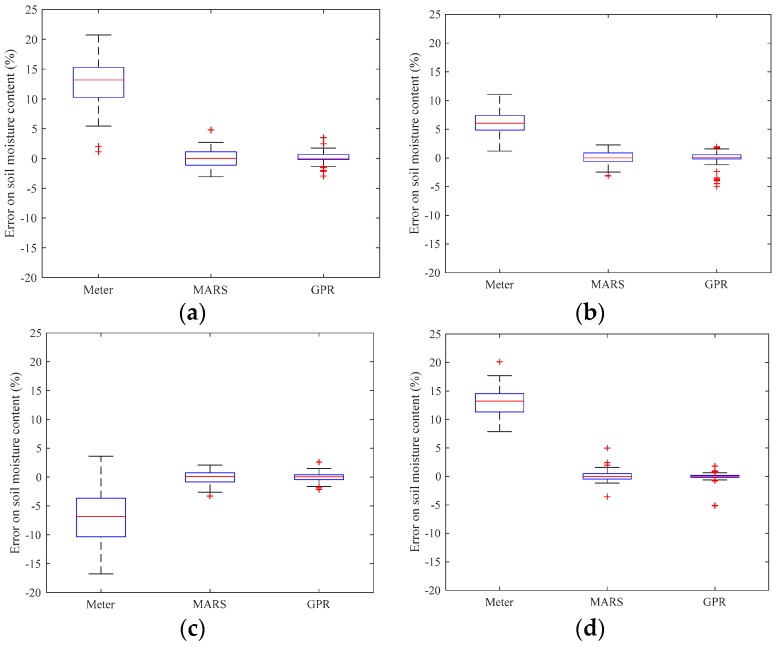
Box plots of the bias error: (**a**) sensor A; (**b**) sensor B; (**c**) sensor C; and (**d**) sensor D.

The boxplot of the bias error is illustrated in Figure 3. It is further demonstrated that the proposed methods highly improved the accuracy compared to the sensor reading, while the variation of the bias errors was also reduced.

**Figure 4:**
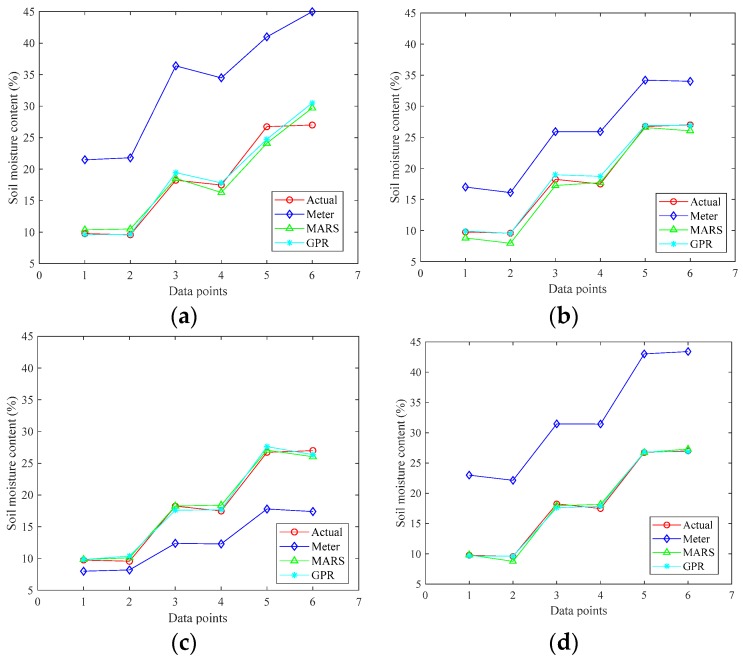
The comparison of soil moisture content by different approaches: (**a**) sensor A; (**b**) sensor B; (**c**) sensor C; and (**d**) sensor D.

To further demonstrate the performance of the proposed methods, the calibrated and actual soil moisture at temperature 30 °C is depicted in Figure 4. It is observable that the modeling soil moisture by the proposed data-driven calibration methods agreed well the actual soil moisture.

From the above analysis, the proposed data-driven calibration of soil moisture sensors considering the impact of temperature can greatly improve the accuracy of soil moisture content measurement. The MARS and GPR model were used due to their strong capability in nonlinear modeling with a limited training dataset. The MARS model can be more efficiently implemented on embedded devices compared to the GPR model in terms of model complexity, while the latter achieved better performance for most cases in this study. Hence, the trade-off between the modeling accuracy and the ease of model implementation should be considered when selecting calibration models in practice. In the future, more machine learning algorithms such as boosted regression trees and neural networks [20] can also be applied to sensor calibration with rich data.

## 5. Conclusions

In this paper, data-driven methods based on the multivariate adaptive regression splines (MARS) and Gaussian process regression (GPR) models were developed to calibrate soil moisture sensors considering the impact of temperature. The effectiveness and efficiency of the proposed method were verified on various soil moisture sensors that belong to the frequency domain reflection (FDR) type. The numerical results demonstrate that the proposed methods can greatly reduce the measurement errors. This study supports the application of data-driven models for the calibration of soil moisture sensors to improve the measurement accuracy for the considered sensors.

## Figures and Tables

**Table 1:** Specifications of the sensors.

Sensor	Type	Structure	Range (v/v)	Working Temperature (°C)
A	FDR	Probe	0–100%	−20–60
B	FDR	Probe	0–100%	−20–60
C	FDR	Tube	0–100%	−20–60
D	FDR	Tube	0–100%	−20–60

**Table 2:** Soil characteristics parameters.

Soil Type	Sand Content (%)	Silty Content (%)	Clay Content (%)	Dry Bulk Density (g/cm^3^)	Withering Coefficient (v/v)	Field Capacity (v/v)	Saturated Water Content (v/v)
Sandy Clay Loam	54.12	24.00	21.88	1.40	17.47%	26.34%	38.92%

**Table 3:** Examples of sensor readings under various temperatures.

*T* (°C)	Sensor (v/v)	Actual (v/v)
0	11.80%	9.77%
5	31.00%	18.25%
10	32.50%	18.25%
15	33.30%	18.25%

**Table 4:** Gaussian process regression (GPR) model parameters.

Parameter	Sensor A	Sensor B	Sensor C	Sensor D
*δ*	0.0028	0.0118	0.0068	0.0028
*σ_f_*	0.0688	0.1159	0.1193	0.0767
λ*_T_*	25.0311	67.0012	33.0755	46.1573
λ*_r_*	0.0356	0.1144	0.0630	0.0480

**Table 5:** Modeling performance comparison (the best values are in bold).

Sensor	Method	MBE (%)	MAE (%)	RMSE (%)
A	Reading	12.93	12.93	13.58
	MARS-1	2.95 × 10^−3^	1.21	1.48
	MARS-2	2.92 × 10^−3^	2.08	2.89
	GPR-1	00.15	**0.70**	**1.03**
	GPR-2	**−1.15 × 10^−3^**	2.07	2.73
B	Reading	6.14	6.14	6.48
	MARS-1	**−2.24 × 10^−3^**	**0.83**	**1.06**
	MARS-2	−9.13 × 10^−3^	1.17	1.92
	GPR-1	−0.14	**0.83**	1.40
	GPR-2	−0.16	1.15	1.93
C	Reading	−7.11	7.49	8.80
	MARS-1	−3.48 × 10^−3^	0.80	1.02
	MARS-2	−7.00 × 10^−3^	4.26	5.42
	GPR-1	4.71 × 10^−3^	**0.57**	**0.78**
	GPR-2	**−1.16 × 10^−3^**	4.40	5.48
D	Reading	13.12	13.12	13.36
	MARS-1	4.96 × 10^−3^	0.65	1.02
	MARS-2	**3.75 × 10^−3^**	0.84	1.37
	GPR-1	−0.12	**0.44**	**0.96**
	GPR-2	−0.28	0.91	1.98

MARS-1: Multivariate adaptive regression splines (MARS) model considering temperature impacts. MARS-2: MARS model without the consideration of temperature impacts. GPR-1: GPR model considering temperature impacts. GPR-2: GPR model without the consideration of temperature impacts.

**Table 6:** The root mean square error (RMSE) in percentages at various temperatures on sensor C.

Method	0 °C	10 °C	20 °C	30 °C	40 °C
Reading	12.05	10.70	8.92	6.30	2.80
MARS-1	1.19	0.55	0.84	0.60	0.97
MARS-2	6.02	4.40	3.07	3.72	7.24
GPR-1	0.28	0.54	0.78	0.62	0.94
GPR-2	5.99	4.54	3.37	3.62	7.41
